# The Prospect of Hepatic Decellularized Extracellular Matrix as a Bioink for Liver 3D Bioprinting

**DOI:** 10.3390/biom14081019

**Published:** 2024-08-16

**Authors:** Wen Shi, Zhe Zhang, Xiaohong Wang

**Affiliations:** 1 Center of 3D Printing & Organ Manufacturing, School of Intelligent Medicine, China Medical University, Shenyang 110122, China; wshi@cmu.edu.cn; 2 Department of Ultrasound, The First Hospital of China Medical University, Shenyang 110001, China; 3 Department of Thoracic Surgery, The First Hospital of China Medical University, Shenyang 110001, China; 20181074@cmu.edu.cn

**Keywords:** biomaterials, 3D bioprinting, decellularized extracellular matrix (dECM), bioink, organoid

## Abstract

The incidence of liver diseases is high worldwide. Many factors can cause liver fibrosis, which in turn can lead to liver cirrhosis and even liver cancer. Due to the shortage of donor organs, immunosuppression, and other factors, only a few patients are able to undergo liver transplantation. Therefore, how to construct a bioartificial liver that can be transplanted has become a global research hotspot. With the rapid development of three-dimensional (3D) bioprinting in the field of tissue engineering and regenerative medicine, researchers have tried to use various 3D bioprinting technologies to construct bioartificial livers in vitro. In terms of the choice of bioinks, liver decellularized extracellular matrix (dECM) has many advantages over other materials for cell-laden hydrogel in 3D bioprinting. This review mainly summarizes the acquisition of liver dECM and its application in liver 3D bioprinting as a bioink with respect to availability, printability, and biocompatibility in many aspects and puts forward the current challenges and prospects.

## 1. Introduction

The liver is a vital organ and has a variety of functions, such as synthesizing proteins and coagulation factors, participating in the metabolism of various substances such as glucose and fat, detoxification, and so on [1].

However, liver disease is prevalent and is responsible for 2 million deaths annually, or 4% of all deaths (1 in 25 deaths worldwide) [2]. Many factors can cause liver fibrosis, such as viral hepatitis, alcohol abuse, primary biliary cirrhosis, autoimmune diseases, and so on [3]. Liver fibrosis is the outcome of excessive accumulation of extracellular matrix (ECM) proteins. Progression of liver fibrosis can lead to liver cirrhosis and liver cancer [4]. Although some studies have suggested the possibility of reversibility, liver fibrosis can eventually lead to irreversible cirrhosis if the injury persists for a long time [5]. In the end, liver transplantation is often the only solution. Due to the severe shortage of donor organs, immuno-suppression, and organ rejection, only a small proportion of patients underwent liver transplantation. Therefore, it is urgent to find substitutes for liver transplantation and construct bioartificial livers in vitro [6].

Over the past few decades, researchers in the fields of tissue engineering and regenerative medicine have been working hard to create bioartificial tissues and organs that can be used for transplantation. Many have turned to the use of decellularized extracellular matrix (dECM) as a scaffold to carry cells for transplantation. Originally, dECM had a natural three-dimensional (3D) structure and a variety of bioactive components, mimicking an optimal non-immune environment [7]. Recently, decellularization techniques have developed rapidly in the field of biomedicine and are widely used in many organs [8]. Subsequent cell seeding construction using dECM as a scaffold material, especially using stem cell recellularized scaffold material, is considered to be ideal for functional organ or tissue regeneration. dECM-derived hydrogels are widely used to reconstruct the cellular microenvironment in tissue engineering and regenerative medicine. For example, Hussein et al. demonstrated in vitro that liver dECM-derived hydrogel could improve human hepatic stellate cell line (LX-2) activation by blocking the transforming growth factor-β1 (TGF-β1)/Smad pathway [9]. Moreover, in vivo experiments showed that the hydrogel could reduce liver fibrosis and restore the liver to a nearly normal structure. Thus, they concluded that hepatic dECM hydrogel could reduce liver fibrosis.

As an emerging technology in the field of bioengineering, 3D bioprinting has totally changed the fate of traditional tissue engineering protocols. In particular, multi-nozzle extrusion-based 3D bioprinting can simultaneously print multiple biomaterials and cell types to mimic their natural counterparts [10]. Multi-nozzle extrusion-based 3D bioprinting can spatially and precisely arrange cells, growth factors, and polymeric biomaterials to form tissue-like 3D structures, which can be used to construct complex tissues and organs in vitro directly [11,12]. So, it holds promise to solve the global problem of organ donor shortage in organ transplantation. Researchers have sought to develop a hepatic bioink that is non-toxic, has adjustable stiffness, and is highly printable. Hepatic dECM bioinks meet all the above requirements and are expected to become an ideal bioink for liver 3D bioprinting. When hepatic dECM bioinks are combined with 3D bioprinting technologies, many other aspects, such as the manufacture of liver organoids and the transplantation of bioartificial livers, will enter a whole new era.

This review introduces the latest research progress of hepatic dECM, 3D bioprinting, and liver 3D bioprinting, the combination of liver 3D bioprinting and dECM, and the application of liver 3D bioprinting in other fields.

## 2. Structure and Function of the Liver Extracellular Matrix

In a healthy liver, the ECM accounts for 10% of the total volume. It plays an important role in regulating tissue homeostasis, remodeling, and regeneration by acting as a structural support with mechanical elastic properties and providing environmental cues that affect cell survival, proliferation, morphology, migration, and differentiation [13].

The ECM is rich in a variety of proteins, which shape the overall structure of organs. It not only contains various proteins but also binds many related molecules, including cytokines, growth factors, and chemokines. Proteins are broadly divided into two main groups: fibrin (such as collagen, fibronectin, and laminin) and glycoproteins (such as proteoglycan).

When the liver is damaged, and liver fibrosis occurs, the balance between synthesis and degradation of connective tissue components is disrupted. Hepatic stellate cells (HSCs) are activated from a resting state into contractile myofibroblast-like cells and secrete excessive amounts of ECM proteins, especially collagen. The metalloproteases released by macrophages are unable to degrade the excess protein. Fibrogenesis is more than fibrolysis. So, the collagen is deposited in the ECM [14].

Remodeling and accumulation of ECM during liver fibrosis leads to a progressive increase in liver stiffness. In addition, increased liver stiffness also promotes the progression of liver fibrosis itself.

Many growth factors, cytokines, and chemokines are present in the ECM, forming a reservoir of biologically active immunomodulatory molecules. For example, transforming growth factor-β1 (TGF-β1) can regulate the homeostasis of the extracellular matrix [15]. During fibrosis, excess TGF-β1 activates HSCs and induces their transdifferentiation into myofibroblasts, which further deposit ECM proteins. TGF-β1 also promotes ECM deposition by amplifying hepatocyte death. Moreover, TGF-β1 exerts immunosuppressive effects by inducing macrophages and impairing the activity of dendritic cell (DC) and natural killer (NK) cells [16].

dECM has been obtained from many animal tissues, such as pigs, cattle, goats, rats, etc. It has also been reported to be obtained from humans [17]. Many organs can be the source of dECM, such as adipose tissue [18], heart [19], liver [20], skin [21], etc. However, human-derived dECM is difficult to obtain, and most of them are probably derived from tissue biopsy or surgical specimens. Therefore, finding animal-derived dECM as a substitute has become a research goal of scientists. Due to the advantages pigs have, such as high reproductive potential, rapid growth, and closer genetic similarity to humans, pig-derived organic dECM is more popular among researchers.

The decellularization process can be performed using chemical agents (such as acids, bases, and detergents), biological agents (such as enzymes and chelating agents), physical methods (such as temperature and pressure), or a combination of the above two or several methods [22]. The proportion of ECM components varies in different tissues, and there are some differences in the decellularization process [23].

In terms of physical and mechanical properties, Ijima et al. found that dECM had lower elasticity compared to type I collagen, and its elastic modulus decreased suddenly at 37 °C [24]. An increase in the concentration of dECM gel results in a decrease in the rate of biodegradation and an increase in mechanical strength. The performance of the dECM gel (10 mg/mL) was comparable to that of the collagen gel (3 mg/mL) [25].

The stiffness of the ECM can affect cell morphology. In a stiffer ECM, cells are subjected to greater traction and, therefore, become increasingly dispersed. However, in a softer ECM, the cell morphology is more close to circular. The stiffness of the ECM also affects cell proliferation, and cells in the ECM with higher stiffness have a stronger proliferation capability. It is believed that soft ECM (<100 kPa) helps to preserve the versatility of embryonic stem cells (ESCs) and induces pluripotent stem cells (iPSCs) [26].

The proportion in the ECM of various tissues also determines the different mechanical properties of natural materials; for example, the ECM of cartilage has a larger storage modulus than that of adipose or heart tissue. In addition, the mechanical stiffness of liver dECM can be improved by chemical modification with the addition of different “polymer backbones” and/or cross-linkers. By doing so, it is possible to alter the mechanical properties without changing the biochemical concentration of liver dECM [27].

There have been some reports reflecting the relationships between the dECM hydrogels and their original organs. For instance, Berger et al. demonstrated clear differences in ECM characteristics between decellularized organs and showed that dECM drives spontaneous differentiation of human induced pluripotent stem cells (hiPSCs) into different lineages in an organ-dependent manner, revealing that dECM hydrogel is organ-specific [28].

Jaramillo et al. used an established liver differentiation protocol to differentiate hiPSC on decellularized human liver matrix (hDLM) scaffolds [29]. The experiments indicated that the use of hDLM resulted in the upregulation of functional liver markers compared to standard differentiation conditions. Cells generated by hDLM expressed more mature hepatocyte markers than Matrigel or type I collagen. Meanwhile, these cells showed similar levels of liver function as neonatal primary hepatocytes. hDLM was most effective when introduced early in differentiation. However, there are some problems in the study. The most important issue is that the cells are still immature, which are not suitable for clinical use. Another issue is the heterogeneity of the hDLM origin.

The study by Hou et al. found that dECM-based biomaterials could ameliorate hepatocyte damage induced by hepatotoxic substances [30]. Exclusive action was performed by infusing dECM into the injury-induced fibrotic liver through the portal vein. The results showed that Glt-HPA-DLM prepared by dECM could enhance liver function, and it was a good matrix to simulate the actual physiological environment of hepatocytes. Although dECM-based biomaterials provide better attachment structures for hepatocytes, hepatocytes are unable to maintain a stable growth rate over long periods of time and even lose their viability and function after 5 days of culture.

Furthermore, Ahmed et al. found that a mixture of liver dECM hydrogel-silver nanoparticles (AgNPs) held the potential to effectively affect the treatment and regeneration of thioacetamide (TAA)-induced liver injury [31]. The dECM hydrogel they prepared could persistently release the ECM components, including growth factors and loaded AgNPs.

dECM biomaterials can be applied in many ways, such as maintaining the morphology of the original tissue and serving as a scaffold for recellularization, injectable hydrogel forms, and patch type, directly used as cell culture substrates. For example, dECM has been used in several forms for cardiac therapy. It can be either solid scaffolds that maintain native vasculature structures or soluble materials that can form injectable hydrogels for tissue repair. Solid scaffolds include tissue-engineered dECM patches/sheets and whole hearts. Soluble materials include injectable hydrogels, 2D and 3D hydrogels, and combinatorial patches [32].

## 3. Liver dECM Scaffolds

**Figure 1:**
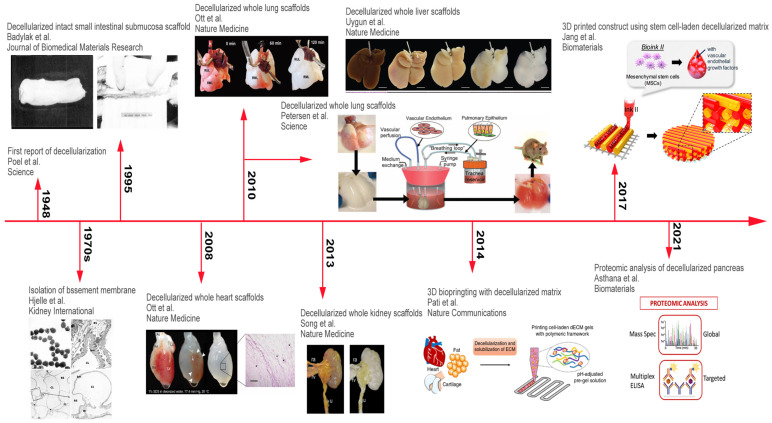
Timeline of dECM scaffolds. The original decellularization technique appeared in 1948 [34], but it was not until the 1970s that the generation of tissue-specific ECM was first reported [35]. Complete decellularized small intestinal submucosa matrices for Achilles tendon repair were pro-duced in 1995 [36]. Whole rat hearts were first decellularized in 2008 [37], and lungs and livers were decellularized in 2010, respectively [38,39,40]. Decellularized kidneys were transplanted in situ in 2013 [41]. Starting in 2014 [42], dECM hydrogels were used for 3D bioprinting and then loaded with stem cells in 2017 [43]. In recent years, dECM materials have been analyzed using a variety of proteomics approaches [44]. Reproduced with permission from [7], Bioactive Materials; published by Elsevier, 2021.With the unremitting efforts of researchers, dECM scaffolds are expected to be implanted into patients through recellularization in the near future and play a role in repairing damaged organs, regenerating endogenous tissues, and replacing missing organs. Scale bar, 200 μm. The area indicated by the arrow in the figure is the left ventricle. * H&E staining of thin section of SDS-treated heart showing no intact cells or nuclei.

In the field of tissue engineering, polymeric biological scaffolds have been developed as a key factor for tissue construction. As with other biodegradable polymers, dECM can also be used as a scaffold material, with good biocompatibility and low immunogenic activity [33]. During decellularization, cells and immunogenic molecules are largely removed [8]. Since the concept of decellularization was proposed, researchers have conducted a lot of exploration on the application of dECM scaffolds. Figure 1 shows the timeline of dECM scaffolds [7].

Decellularized scaffolds derived from animal organs are currently being explored as promising resources for generating transplantable and functional organs. Many researchers have considered bioengineered livers generated from decellularized whole liver scaffolds and iPSC-derived hepatocytes (iPSC-Heps) as an alternative method for the therapy of end-stage liver disease [45].

Currently, the cells that are commonly used are primary hepatocytes. However, primary hepatocytes have limited expansion capacity and are prone to immune rejection in the early stage of transplantation [46]. In recent years, researchers have tried recellularization with other cells. Stem cells are becoming an alternative to primary hepatocytes due to their ability to renew and differentiate into various cells. For example, induced human pluripotent stem cell-derived hepatocytes constitute an unlimited source of cells and are less immunogenic [47]. Guan et al. have demonstrated the chemical reprogramming of human somatic cells into human chemically induced pluripotent stem cells that exhibit key features of embryonic stem cells by creating an intermediate state of plasticity [48]. Therefore, it is expected to induce patient-specific pluripotent stem cells through reprogramming, which will pave the way for personalized diagnosis and treatment and precision medicine.

Obtaining a dECM scaffold of the liver mainly includes physical, chemical, and enzymatic treatments. Table 1 summarizes the mechanisms, advantages, and disadvantages of various decellularization methods. Commonly used physical methods include freeze-thaw cycles, perfusion, immersion, and agitation. Physical treatments regulate physical properties such as temperature, pressure, and other factors to facilitate the washing of detergent solutions, destruction of cell membranes, and removal of cell contents. Among them, immersion and agitation were applied when the thickness of the liver slice was <5 mm [49].

Commonly used chemical reagents include ionic and non-ionic detergents, acids and bases, and hypertonic and hypotonic solutions. Sodium dodecyl sulfate (SDS) is the most widely used ionic detergent that lyses cell membranes and denatures proteins. Triton-X-100, the most commonly used non-ionic detergent, disrupts lipid-lipid, lipid-protein, and DNA-protein interactions and is gentler than SDS. Although SDS was more efficient than Triton-X-100, more glycosaminoglycan and elastin proteins were destroyed. However, Triton-X-100 could retain more dECM. As a kind of bioink, the higher the dECM content, the better the performance of 3D bioprinting [50,51,52]. While ensuring the effect of decellularization, attention should also be paid to reducing the residue of detergent and reducing the formation of thrombosis and cytotoxicity after recellularization and implantation [53].

Commonly used enzymes include nuclease, trypsin, dispase, lipase, and collagenase. Among them, nuclease and trypsin are the most commonly used. Enzymes can remove cellular components, improve the decellularization effect of detergents, and prevent adverse immune reactions later.

Physical, chemical, and enzymatic treatment methods each have advantages and disadvantages. So, these methods are often used in combination to maximize their respective advantages and minimize ECM destruction. In addition to the several commonly used methods mentioned above, several new therapeutic approaches have recently emerged, such as vacuum-assisted decellularization and apoptosis-assisted decellularization [54,55]. However, these new methods have not been widely used and are still worth further exploration in the future.

The quality of the liver decellularized scaffold is critical for the following recellularization and transplantation. However, there is currently no unified standard for assessing the quality of the decellularization. Currently, the extent of decellularization is mainly determined by residual DNA. The generally acknowledged criteria are as follows: (1) DNA content < 50 ng/mg dry ECM weight; (2) DNA fragment length < 200 base pairs; (3) lack of visible nuclear content with DAPI and HE staining [56]. Most qualitative and semi-quantitative studies employ morphological and molecular features to evaluate decellularized scaffolds. Morphological evaluation was performed using the appearance of the scaffold, HE staining, and SEM to assess its remaining microanatomy and 3D structure. Molecular evaluation, including residual DNA, cell factors, and quantitative evaluation of the ECM proteins.

With the above treatments, dECM liver scaffold can remove immunogenic cellular components while retaining important components and natural structure, providing a microenvironment for subsequent tissue repair, recovery, and regeneration.

dECM liver scaffolds are then subjected to post-processing, including sterilization, cross-linking, and modification. After post-processing, dECM liver scaffolds also need to be recellularized.

Recellularization refers to the proliferation of cell-free ECM scaffolds with specific cell types or stem cells to form engineered constructs with specific structures and functions. Pluripotent stem cells with multiple differentiation potentials are ideal choices for recellularization. Pluripotent stem cells include ESCs and iPSCs. Compared with ESCs, iPSCs have fewer ethical issues and a wide range of sources, such as bone marrow, adipose tissue, and central nervous system (CNS).

Moreover, recellularization is closely related to cell types, methods of recellularization, cell density, and culture conditions [57]. The liver cannot be recellularized to perform complex liver functions with only one cell type. Decellularized scaffolds must be systematically infused with various cells in the correct positions.

The liver dECM scaffold has a portal vein, hepatic artery, hepatic vein vascular network, and complex biliary system. Cholangiocytes need to enter the liver through the common bile duct, and hepatocytes need to be perfused into the liver from the portal vein and settle in the liver parenchyma.

In terms of the source of decellularized scaffolds, many studies have used porcine decellularized scaffolds due to the shortage of discarded human livers [58,59]. The anatomical structure of the porcine liver is similar to that of humans, and it is suitable for size and has a long life span, which is an ideal model for preclinical research. Cells can colonize, proliferate, and differentiate.

The microenvironment of ECM can regulate stem cell self-renewal and lineage commitment [60]. Since human Pluripotent stem cells (hPSCs) require organ-specific ECM to drive phenotypic differentiation and maturation in a 3D culture environment, Goh et al. used ECM from decellularized organs, namely pancreas, liver, and heart, to construct an ECM array platform that allows cell-ECM interactions in 2D and 3D configurations [61]. They tested the platform by elucidating the role of three different organ-specific ECMs in supporting the induced pancreatic differentiation of hPSCs. The platform they developed can be used to characterize the specific differentiation of any lineage.

Park et al. reimplanted iPSC-Heps into decellularized liver scaffolds and cultured decellularized livers using a continuous perfusion system [45]. After the short-term transplantation of recellularized liver scaffolds into rats, the grafts expressed hepatocyte markers and did not rupture. Their results showed that more than 40 different liver-derived growth factors were preserved after decellularization. They suggested that although the role of dECM in the liver maturation of iPSCs is not fully understood, liver-derived growth factors and ECM may influence liver development and maturation. And dECM may also be useful for liver differentiation of human iPSCs because ECM components and functions are highly conserved in different species [62]. Moreover, the xenogeneic source of ECM does not significantly affect the survival and function of the repopulated cells. These results lay the foundation for the development of bioengineered livers using stem cells and decellularized scaffolds.

In addition to recellularization, liver dECM can also play a role in liver tissue engineering by binding proteins or macromolecules. For example, Sasikumar et al. reported the modification of liver dECM by the addition of galactose residues. Compared with the liver dECM undecorated, galactose residue modification can enhance the specificity of the HepG2 metabolism, build better cell polarity, and improve the function of HepG2 cells [63].

Currently, liver dECM scaffolds still have many limitations. For example, only cells at the edge of the scaffold survived due to the restricted blood supply to the whole area. Furthermore, insufficient cell numbers remain a challenge, and generating clinically sufficient amounts of cells is costly. In addition, iPSC-Heps have limited proliferation and long-term survival capacity. Thus, the development of patient-specific stem-cell-derived hepatocytes that have a long lifespan and are scalable is needed.

We also need to think about how to avoid thrombosis. Long-term in vivo transplantation of bioengineered organs requires more effective anticoagulation strategies. Although the dECM scaffold retained the intact vascular network, the endothelial layer was lost [64]. Collagen in the ECM triggers platelet activation, aggregation, and the production of thrombin and fibrin, which triggers thrombus formation. Therefore, the long-term survival of liver implants is hindered.

There are several limitations to the use of decellularized ECM scaffolds in in vitro models. Compared with natural scaffold, it has limited mechanical instability and significant changes in mechanical properties. Interbatch variation due to differences in decellularization processes between donor age, sex, genetics, treatment, and anatomical tissue sampling. For example, the liver of older adults tends to contain more total collagen, sulfated glycosaminoglycans (GAGs), and fewer growth factors. Therefore, donor age is a key factor affecting the quality of the new liver generated by the liver dECM scaffold [65]. Other limitations include the inevitable removal of some growth factors and ECM proteins from the matrix and the difficulty of quantifying the exact protein content in different ECMs.

The most important problems to be solved in decellularized scaffolds are the loss of some ECM components, ultrastructural changes, and poor cell attachment or recellularization after decellularization [66].

Many attempts have been made to solve some of the problems existing in liver-decellularized scaffolds. Researchers found that loading prednisolone into a liver dECM scaffold could be a valuable method to limit inflammation after transplantation [67]. Although the combined mesenchymal stem cells/prednisone preloaded scaffold did not alleviate inflammation, it played an important role in regeneration and angiogenesis.

## 4. 3D Bioprinting and Liver 3D Bioprinting

### 4.1. 3D Bioprinting

3D bioprinting enables the fabrication of complex 3D geometries containing multiple cell types and different biomaterials. It offers solutions for the accurate deposition of various biomaterials and multiple cell types, as well as for the fabrication of biological structures in vitro. The formation of 3D biological structures is achieved by a bottom-up assembly in which biomaterials and cells are deposited layer by layer in a predefined pattern.

3D bioprinting can be used with human cells, so it may better reflect the physiological and pathological situation of humans than animal experiments, thus helping to overcome some of the shortcomings of animal models. The quality of 3D bioprinted scaffolds can be affected by many factors, such as biocompatibility, degradation time, tissue microenvironment, and so on.

**Figure 2:**
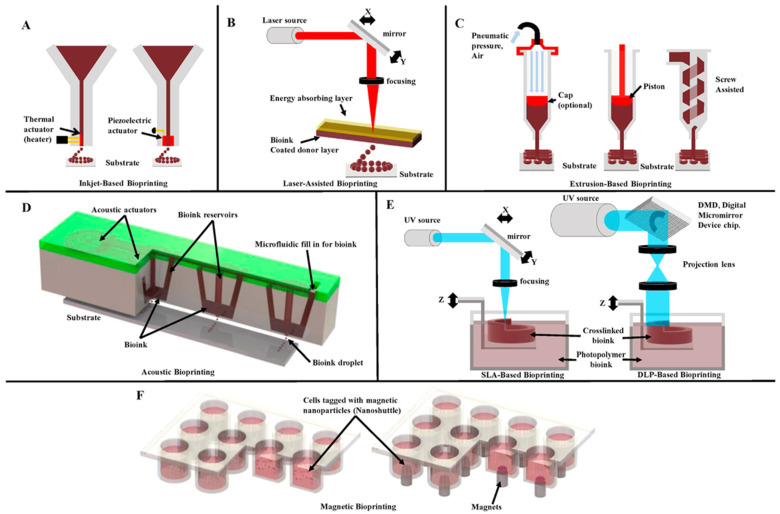
Working principle of several 3D bioprinting modalities. (**A**) Inkjet bioprinting systems, including thermal and piezoelectric Drop-On-Demand (DOD) based mechanisms. (**B**) Laser-assisted bioprinting system; Laser-induced forward transfer mechanism. (**C**) Extrusion-based bioprinting systems include pneumatic pressure, piston, and screw-assisted mechanisms. (**D**) Acoustic bioprinting system; a type of DOD mechanism. (**E**) Stereolithography bioprinting systems, including SLA and DLP (Digital Light Processing) laser-based mechanisms. (**F**) In a magnetic bioprinting system, cells are shown within the culture media. Reproduced with permission from [68], Biomaterials; published by Elsevier, 2020.

According to different working principles, 3D bioprinting can be roughly divided into the following: inkjet-based bioprinting, laser-based bioprinting, extrusion-based bioprinting, acoustic bioprinting, vat photopolymerization bioprinting (VPB) and magnetic bioprinting [68,69]. These bioprinting methods can be used alone or in combination. Figure 2 illustrates the working principle of several 3D bioprinting modalities [68].

Among them, extrusion-based bioprinting is widely used, relatively economical and simple. However, cells may be damaged by the pressure during printing, resulting in reduced survival rate and impaired function of the printed tissue. Therefore, the effect of extrusion-based bioprinting can be improved by adjusting the shear stress to change the printing parameters, such as pore size, layer thickness, and angular pattern [70].

Laser-based bioprinting is also widely used. It is based on cutting techniques that can create precise structures with extremely high resolution. However, the range of materials that can be used for laser-based bioprinting is limited, and the laser wavelengths used can damage cells, resulting in reduced survival, and the cost is high.

Inkjet-based bioprinting is performed by deforming the print head using a thermal or piezoelectric actuator to produce a droplet of adjustable size from the bioink stored in the cartridge in a continuous manner. Inkjet-based bioprinting can achieve high-resolution printing with fast printing speed. However, the principle of inkjet-based bioprinting limits the selection of bioinks, which can only print materials with low viscosity due to the configuration of the nozzle. Moreover, the size of bioprinted constructs is limited, and the cost is relatively high [71].

VPB is mainly divided into three subtypes, including stereolithography (SLA), digital light processing (DLP), and two-photon polymerization (2PP) [72]. SLA-based bioprinting uses a laser to irradiate a portion of a photo-crosslinked bioink called resin in a point-by-point fashion, curing it into the desired structure. DLP-based bioprinting differs from SLA in that instead of using a laser beam, a liquid crystal display (LCD) or a digital micromirror device (DMD) is used to project the full image onto the resin layer. 2PP bioprinting uses a femtosecond laser to act on the photoinitiator (PI) molecule, causing it to absorb photons and initiate free-radical polymerization at the focus of the laser beam, thereby forming a solidified structure to realize the cross-linking of bioink [73]. VPB bioprinting has high resolution and can create complex structures, but it can damage cells when exposed to ultraviolet light and is expensive. Each bioprinting technique has its own characteristics and advantages, and the specific technique chosen depends on the needs of the printed tissue.

A crucial component of 3D bioprinting is bioink, which is printed with living cells during the printing process. It is a liquid containing nutrients, ECM components, and various arranged cells [74]. Bioinks can be fabricated into 3D simulated tissue structures with geometric complexity. This allows the creation of new functional 3D tissues from cellular sources.

Bioinks can be divided into 4 types based on their effects: (1) Structural bioinks support cell proliferation, differentiation, and adhesion, can mimic the extracellular matrix, and maintain mechanical integrity. (2) Fugitive bioinks or sacrificial bioinks are temporary materials that can be quickly removed to create internal voids or channels in the 3D-printed structure. (3) Support-bioinks are non-biological materials with good mechanical strength that can supply mechanical support for complex structures or softer materials during printing. (4) Functional bioinks can offer biochemical, mechanical, and electrical signals to affect cell behavior after bioprinting [56]. The rheological, biological, and mechanical properties of bioinks can determine the function of printed tissues and organs.

The ideal bioink should have the following characteristics: good biocompatibility, curing in a method that does not harm cells, printability, good mechanical stability, and the ability to support and facilitate cell activities such as cell proliferation, differentiation, and migration [75].

The polymers used in bioinks can be natural, synthetic, or a combination of both, depending on the type of material. The natural polymers used for bioinks include alginate, collagen, gelatin, fibrin, agarose, chitosan, hyaluronic acid (HA), etc. Scaffolds printed individually from these natural materials usually have low viscosity and are not suitable for direct implantation and reconstruction of weight-bearing tissues. The synthetic polymers used for bioinks include polyethylene glycol (PEG), polyurethane (PU), polylactic acid (PLA), polyhydroxybutyrate (PHB), etc. However, these materials do not represent the complexity of the ECM and are therefore insufficient to reconstruct the microenvironment with cell-ECM junctions and 3D cellular organization. Compared to synthetic hydrogels, dECM hydrogels have better biocompatibility and bioactivity [76]. This may be because some components are retained in the ECM, such as structural and cell adhesion proteins, growth factors, and GAGs. Therefore, dECM is an ideal hydrogel to provide cells with a microenvironment similar to their parent tissue.

Moreover, a scanning electron microscope (SEM) showed that the liver dECM hydrogel maintained a good porosity. So, it was able to absorb large amounts of the surrounding liquid medium, which suggests that hydrogel can be used as a carrier for bioactive molecules, including growth factors, small molecules, or antibiotics. The hydrogel was pre-treated with these bioactive molecules prior to use. After implantation, these molecules would be rapidly absorbed and take effect.

### 4.2. Liver 3D Bioprinting

At present, some studies have reported the application of 3D bioprinting in liver tissue engineering [77]. Compared to a single component of bioink, multicomponent bioink has more advantages, such as improving the printing on the mechanical properties of the tissue construct and cell function [78]. The cells as bioink used for liver 3D bioprinting included liver parenchymal cells (hepatocytes) and non-parenchymal cells (hepatic stellate cells, hepatic sinusoidal endothelial cells, and Kupffer cells). Hepatic parenchymal cells, such as hepatocytes and cholangiocytes, are responsible for the major functions of the liver, such as glucose metabolism, bile synthesis, etc. The non-parenchymal cells mainly play a connecting and supporting role.

Primary hepatocytes, which are directly isolated from the liver, are the ideal cell source for bioprinting to construct liver tissues in vitro due to their high metabolic activity. However, due to the lack of human-derived primary hepatocytes, and these cells can easily lose the phenotype. Researchers have tried to use hepatocellular carcinoma cells, such as HepG2 and HUH7, which have some functions of primary hepatocytes, such as albumin secretion and urea synthesis, so they are expected to be used to construct liver tissues in vitro.

Extrusion-based bioprinting is currently the most widely used method to construct liver tissue scaffolds due to the versatility and ease of handling of bioinks [79]. Extrusion-based bioprinting can be used to print major parts of liver units containing hepatocytes, and the required experimental environment is simple and easy to construct. When embedding microvessels is required, high-resolution DLP technology can create more complex scaffolds that have higher resolution [80]. However, the environment needed for the experiment is also more complex. Therefore, extrusion-based bioprinting is the main method of 3D bioprinting of the liver. Unlike inkjet-based bioprinting, extrusion-based bioprinting produces continuous filaments through nozzle movement to form 3D structures of different shapes, sizes, and resolutions layer by layer.

The vascular system plays a crucial role in liver function, so the construction of the vascular system in liver 3D bioprinting is crucial for the successful construction of liver tissue. At present, there are three main methods to print liver blood vessels: coaxial bioprinting, sacrificial materials, and self-assembly. Coaxial bioprinting uses a coaxial nozzle system to deposit bioink containing both parenchymal and endothelial cells, which creates a functional vascular network. Sacrificial materials refer to the use of sacrificial materials to print with the cells at the time of printing and then to remove, leaving hollow tubes that can be reloaded with endothelial cells to form blood vessels. Self-assembly means that endothelial and supporting cells can self-assemble into functional capillary-like structures. Under suitable culture conditions, it can mimic natural angiogenesis. Among them, coaxial bioprinting is widely used.

However, there are still some problems in printing vascular networks in liver models. For example, the liver vascular network is complex, with portal vein, hepatic artery, and hepatic vein, and the diameter and connection pattern of branches at each level are not the same. Moreover, effective vascular connection between the implanted liver model and the host is also very important. Therefore, solving the vascularization problem of the printed model is crucial for the construction of a 3D-printed liver model.

If the 3D bioprinting model of the liver can be successfully constructed, it can benefit a lot in surgical training and medical education. Surgeons can practice surgery in advance and reduce the failure rate of surgery by using realistic liver models. Medical students can make use of more realistic models to study the liver.

## 5. Liver 3D Bioprinting Combined with Liver dECM

Bioinks are material formulations and biomolecules or cells processed during bioprinting. Synthetic materials such as gelatin, chitosan, and alginate can be used as bioinks. However, these materials do not represent the complexity of the ECM and are therefore insufficient to reconstruct the microenvironment with cell-ECM junctions and 3D cellular organization. Organ-derived dECM bioinks are arguably the most biomimetic bioinks since no material, natural or man-made, can recapitulate all the features of the natural ECM [81].

**Figure 3:**
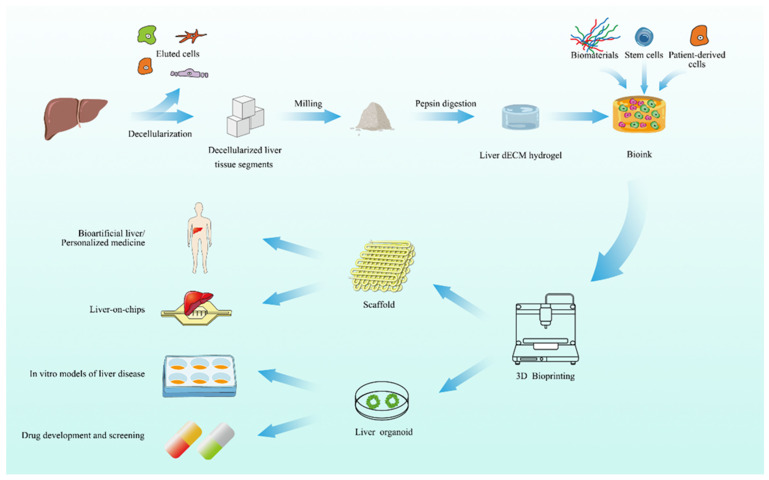
The process of performing 3D bioprinting with liver dECM bioink. After decellularization of the liver, dECM hydrogels can be formed. After that, by adding various biomaterials or cells, bioinks for 3D bioprinting can be formed for a variety of applications.

Currently, dECM bioinks have been used to produce a variety of organs, such as the heart, liver [42], adipose tissue, cartilage, skeletal muscle [82], skin, and vascular tissue. The liver decellularized matrix contains more growth factors involved in cell signaling than conventional bioinks. Shyama et al. found that liver dECM has the potential to preserve the phenotype and function of hepatocytes in the existence of a tissue-specific microenvironment [83]. Studies have found that liver dECM can better promote the proliferation, differentiation, and functional expression of hepatocytes than traditional bioink [84]. Figure 3 shows the process of 3D bioprinting with liver dECM bioink.

After the removal of residual reagents, decellularized tissue can be lyophilized and ground to form a powder that can be dissolved in an acidic environment by pepsin digestion. A gel-like substance can be produced by physical agitation. Digestion can be stopped when the pH of the solution is neutralized to physiological conditions (pH = 7.4). An important advantage of using dECM bioinks for 3D printing is that the pepsin-digested ECM solution is able to act as a bioink by self-assembling into a cross-linked gel at physiological pH levels and temperatures via entropy-driven collagen dynamics [85].

Despite its many advantages, dECM bioink has poor mechanical properties and is softer than other bioinks, so it is often mixed with other crosslinking agents or printed with biocompatible thermoplastics (such as polycaprolactone) to form mechanically strong scaffolds [43]. Zhang et al. summarized some methods used to promote the printability of dECM bioink, including physical strategies and chemical strategies [85].

Kim et al. prepared dECM microparticles with a size of approximately 13.4μm by decellularization of porcine liver and loaded them into the gelatin mixture [86]. The results show that the bio-ink has better mechanical properties, better 3D printing performance, and good cell compatibility.

In addition to mixing it with other materials, sacrificial material is also used. Lewis et al. created specialized structures consisting of dECM by mixing dECM with a sacrificial material, 3D-printed Pluronic F-127 [87]. It has been shown that the encasement of biliary epithelial cells within the dECM of the liver can form a complex bile duct tree in vitro. Thus, by changing several aspects of the dECM geometry structure, such as width and angle, the researchers were able to determine the directional formation of the biliary tree. Their study showed that the constructed 3D structure could not only be used to form an intrahepatic biliary tree but also help to study the cell-cell interactions and bile duct formation. The biliary system is often overlooked in liver 3D bioprinting. However, biliary liver disease is also a main reason for liver failure. If the function of the biliary system is not restored, the liver is unable to secrete and exclude bile even if hepatocytes are present. Therefore, the successful construction of the intrahepatic bile duct tree by Lewis P.L. et al. is of great significance for perfecting the 3D bioprinted structure of the liver.

Lee et al. developed and studied the features and performance of liver dECM bioink for 3D bioprinting [88]. They first evaluated the biochemical properties, printing parameters, rheological properties, and cytotoxicity, as well as printed constructs of liver dECM bioink. Then, the stem cell differentiation and function of HepG2 cells in liver dECM bioink were appraised and compared to cells in commercial collagen bioink. The liver dECM bioink they developed enhanced the function of the printed structure. So, compared to other commercial biomaterials based on liver-specific biochemical residues, liver dECM bioink is expected to better induce liver differentiation and enhance liver-specific functions. They found that choosing the right nozzle size, printing speed, and pressure is important for 3D cell printing of liver dECM bioink. The liver dECM bioink they prepared can be used for liver tissue engineering and liver in vitro models. Moreover, they found that the liver dECM bioink provided a more suitable microenvironment for stem cell differentiation. The expression levels of related genes in the dECM group were higher than those in the collagen group.

The most commonly used technique for printing dECM bioinks has been extrusion-based bioprinting. Extrusion-based bioprinting has many advantages, such as the high density of printed cells and flexible, cost-effective printing of porous scaffolds [89]. By extrusion bioprinting, Hiller et al. added 0.5 and 1 mg/mL hECM to alginate/gelatin bioink, which improved not only the stability of printed constructs but also improved cell viability and metabolic function [90]. The printed 3D liver models supported efficient adenoviral replication and can be used to study viral biology and develop new antiviral drugs.

Although extrusion bioprinting has been widely used in liver tissue engineering, it still has the shortcomings of low printing resolution and poor structural integrity [91,92]. To overcome these problems, some researchers have attempted to utilize photopolymerization-based bioprinting techniques, such as DLP.

Mao et al. developed liver-specific bioinks by combining photocurable methacrylated gelatin (GelMA) with liver dECM and encapsulated human-induced heptocytes (hiHep cells) to form cell-loaded bioinks [93]. In terms of physical properties, this hydrogel was found to have good mechanical, swelling, and degradation properties by measurement. Then, they used DLP-based bioprinting to assemble liver microtissues and found that liver dECM could improve the cell viability and printability of GelMA bioinks, providing a hopeful dECM-based cell-loaded bioink for the fabrication of liver microtissues. The presence of dECM allows hiHep cells to maintain a stable survival state with continuous liver function metabolism, which can be used as a possible treatment for patients with chronic liver failure or partial hepatectomy.

The combination of 3D bioprinting and dECM can be used not only to construct normal liver tissue but also to construct liver disease models.

Liver cirrhosis, even hepatocellular carcinoma (HCC), is an inevitable course in the development of many liver diseases. Therefore, building a suitable liver cirrhosis model is very important in studying the disease. Ma et al. developed photocrosslinkable dECM and a rapid light-based 3D bioprinting process to enable liver dECM with customizable mechanical properties [94]. Their constructed liver dECM scaffold was able to stably reproduce the clinically relevant mechanical properties of cirrhotic tissue. Their results showed that HepG2 cells exhibited growth inhibition and upregulation of invasion markers when encapsulated in dECM scaffolds with cirrhosis stiffness compared to healthy controls. Their results have led to significant advances in rapid 3D graphing of complex ECM biomaterials with biomimetic structures and tunable mechanical properties for in vitro disease modeling.

## 6. Other Application Areas of Liver Bioprinting

3D bioprinting can mimic the human microenvironment in vitro, which makes it superior to the traditional 2D culture mode in many aspects, such as drug screening, transplantation, clinical application of artificial organs, and tumor disease research.

### 6.1. Drug Development and Screening

In order to effectively reduce the morbidity and mortality of liver disease, it is necessary to develop drugs with better efficacy and fewer adverse reactions. Although drug development has created a huge market, there are still many drugs that fail to pass clinical trials. One important reason is that researchers often develop new drugs through 2D cell culture and animal experiments. However, these methods can be costly and time-consuming. Furthermore, 2D monolayer cell culture fails to accurately replicate the metabolic microenvironment of drugs within the human body. Previous drug screening mostly relied on animal experiments, but animal models have limitations due to species differences, ethical issues, and other reasons. Moreover, in some diseases, it is difficult for animal models to mimic human disease progression and predict human response to drug treatment [95,96]. To reduce animal testing, researchers have been trying alternative methods. Three-dimensional bioprinting is expected to be a good option in the future. The researchers hope that 3D bioprinted models can produce the same physiological responses as whole organs on a small scale to test whether drugs are toxic or effective in a complex in vivo environment. Moreover, 3D bioprinting models can better reveal the mechanism and law of disease development than 2D cell culture models, thereby improving the in vitro and in vivo correlation in clinical drug trials.

In drug development and screening, 3D bioprinting can produce models that more closely mimic the physiological processes in vivo and improve the repeatability of the models. Drug efficacy and toxicity can be predicted more accurately.

3D liver models are particularly relevant for drug development because drug-induced liver injury is common in clinical trials [97]. Janani et al. used a novel liver ECM-based bioink and extrusion-based bioprinting to print human adipose-derived mesenchymal stem cells-derived hepatocyte-like cells (HLCs), human umbilical vein endothelial cells (HUVECs), and human hepatic stellate cells (HHSCs) [98]. They performed drug toxicity assessments after exposing the printed liver model to several hepatotoxic drugs at different concentrations for 24 h. Subsequent assessments showed that the hepatotoxic effect was dose-dependent and clinically relevant, as evidenced by changes in cell viability and metabolic capacity. These results confirmed that 3D bioprinted liver models are economically feasible and can be used for drug development and screening.

### 6.2. Cancer Research

3D bioprinting can combine cancer cells and stromal cells into 3D models to generate complex disease models. Moreover, it can customize the composition of the extracellular matrix to generate a specific tumor microenvironment (TME). TME is composed of tumor cells, tumor stromal cells, and ECM. Tumor stromal cells include stromal fibroblasts, endothelial cells, and immune cells such as microglia, macrophages, and lymphocytes [99]. The 3D matrix can be used to reconstruct the main components of TME in an in vitro bioprinted cancer model, which can simulate real tumor cell-cell and cell-ECM interactions [100]. Moreover, it can simulate various stages of tumors and investigate the alterations of a particular molecule. However, 2D culture failed to reproduce the original TME and cell-cell interaction of tumor cells.

Under the action of dECM, cells can better proliferate and differentiate so as to realize the mutual coordination between tumor cells and the environment. Human immune cells can be added to 3D bioprinted tumor models to replace immunodeficient mice. This will not only reduce the need for animal experiments but also reduce species differences and more intuitively observe how tumor cells and immune cells interact.

**Figure 4:**
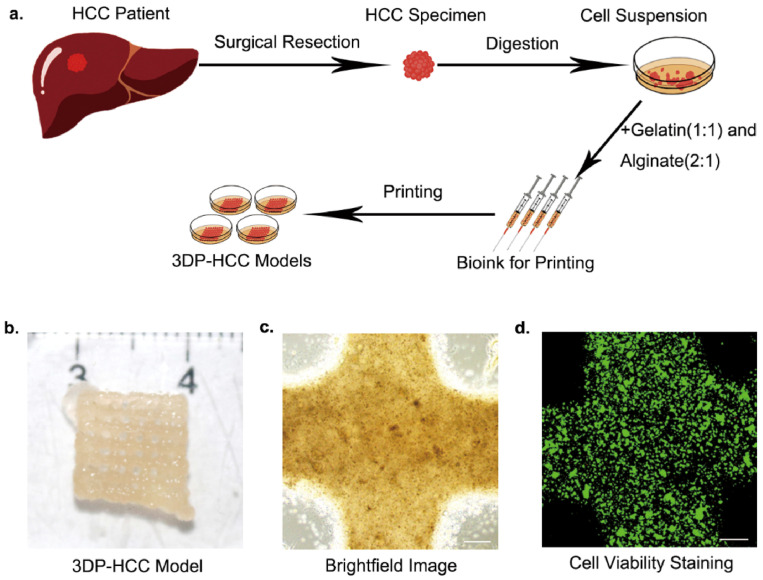
Constructing a clinical patient-derived 3D liver cancer model in vitro using 3D bioprinting. (**a**) Samples obtained by surgical resection are processed to form a suspension of liver cancer cells. By mixing with sodium alginate and gelatin, bioink is formed for 3D bioprinting. (**b**) The general appearance of the printed model. (**c**) A uniform distribution of cells within the model was visible under a light microscope. (**d**) After 1 month of in vitro culture, more than 80% of the HCC cells still maintained good cell viability. Reproduced with permission from [101], Biomaterials; published by Elsevier, 2021.

**Figure 5:**
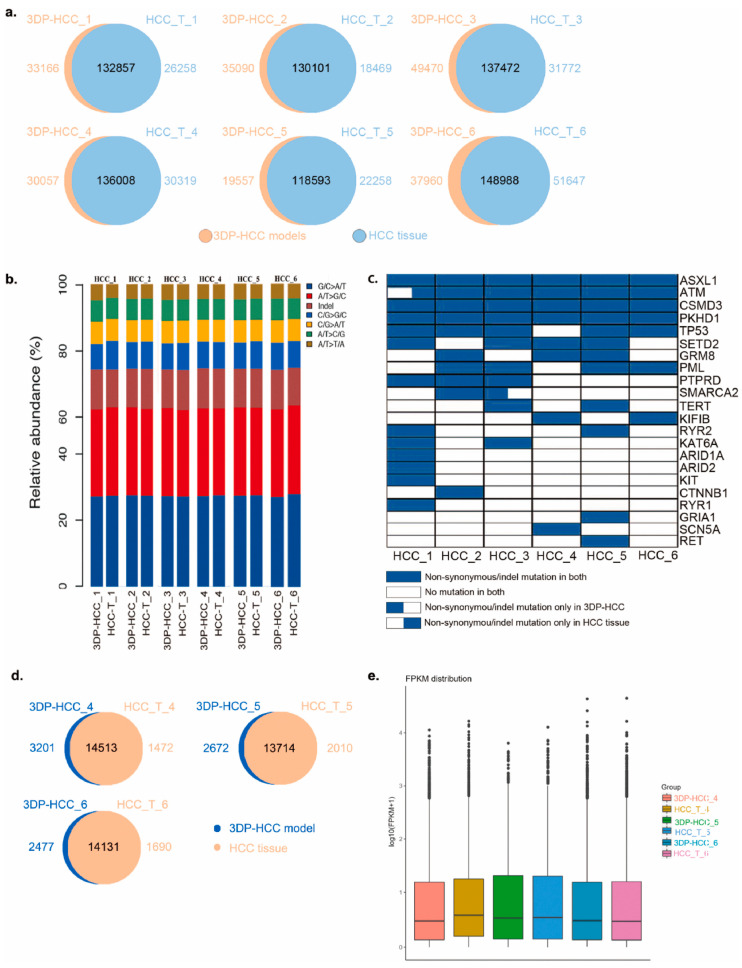
The 3D tumor model cultured in vitro retains the genetic alterations and expression profiles of the original tumor. (**a**) Single nucleotide variants (SNVs) overlap between 3D bioprinting HCC models and parental tumors of the six patients after two weeks of culture. (**b**) Proportions of exonic variants for all samples, the 6 types of SNVs, and indels are represented. (**c**) Mutational profile of the tumor and parental tumors. A total of 22 known HCC driver genes with missense mutations (non-synonymous/indel) are shown on the right. Each row represents a driver gene, and each column represents the mutational profile of 3D bioprinting HCC models and parental tumors. (**d**) Differentially expressed genes overlap between 3D bioprinting HCC models and parental tumors. (**e**) Expression (FPKM scores) distribution of 3D bioprinting HCC models and parental tumors. Reproduced with permission from [101], Biomaterials; published by Elsevier, 2021.

Figure 4 shows how to construct a clinical patient-derived 3D liver cancer model in vitro using 3D bioprinting [101]. Figure 5 shows that the 3D tumor model cultured in vitro retains the genetic alterations and expression profiles of the original tumor [101]. The advantages of 3D bioprinting tumor models include accuracy, reproducibility, and clinical translation. Therefore, 3D tumor models can change the original way of discovering therapeutic targets and developing new drugs and make personalized precision cancer treatment possible.

### 6.3. Organoid

Organoids recapitulate some principles of organ biology in vitro and provide simplified and easily accessible “minimal systems” for identifying the relative contributions of different tissue components to complex morphogenetic processes. Organoid technology can reproduce the cellular heterogeneity, structure, and function of tissues by creating 3D models from stem cells, which has revolutionized in vitro culture tools for biomedical research [102]. Compared with traditional 2D culture and animal models, organoids can more closely mimic the physiological and biochemical characteristics of the human body, including spatial structure, cellular heterogeneity, and cell-cell and cell-ECM interactions [103]. Liver organoids have also been formed by studies including primary hepatocytes, choalngiocytes, and other cell types. Hepatocytes maintain viability and function and become polarized by forming bile canaliculi. Cholangiocytes support the viability and function of hepatocytes in a complex 3D organoid. The bile drainage system for the secreted bile within the hepatic organoid allows the clearance of toxic bile from the cultured hepatocytes’ direct environment.

**Figure 6:**
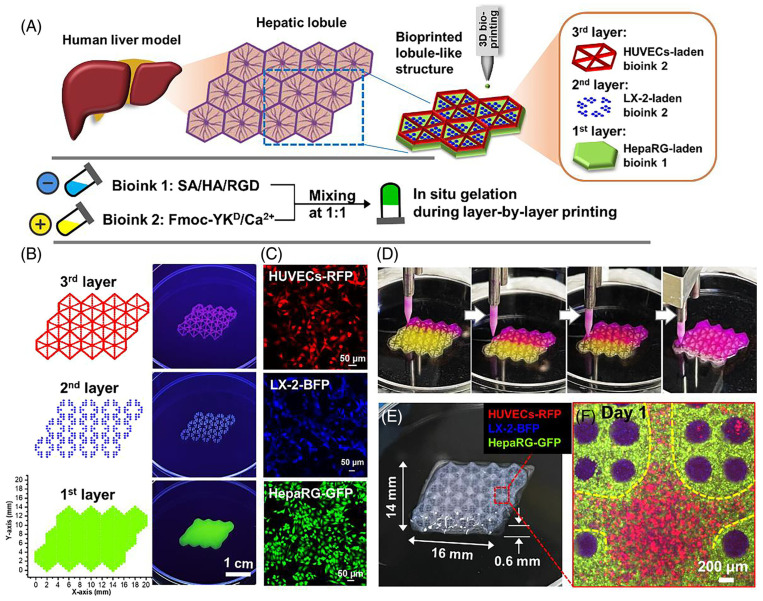
The (**A**) Schematic diagram of liver organoids with biomimetic lobular structure constructed using a multicellular 3D bioprinting strategy. (**B**) Programming of 3D droplet-based bioprinted in vitro liver models. (**C**) Multicellular composition of the constructed liver organoid: red-fluorescent HUVECs (HUVECs-RFP), blue-fluorescent LX-2 (LX-2-BFP), and green-fluorescent HepaRG (HepaRG-GFP). (**D**) Photos of the printing process. (**E**) Optical image of 3D constructed liver organoid with hydrogel scaffold. (**F**) Confocal image of the hexagonal central region in the constructed lobule-like structure after 1 day of culture. Reproduced with permission from [104], Cell Proliferation; published by Wiley, 2023.

As shown in Figure 6, Jian H et al. produced a liver organoid with a bionic liver lobular structure by performing layer-by-layer bioprint using a droplet-based 3D bioprint equipped with a nozzle with an inner- diameter of 190μm [104]. Compared with 2D culture, the cells embedded in liver organoids showed stable proliferation and cell viability at day 7. Moreover, the secretion of ALB and urea of liver organoids was more than 2D culture.

Hepatocytes in 3D-printed liver organoids can come from several different sources, including iPSCs, reprogrammed hepatocytes, and primary hepatocytes [105]. Mun et al. created a novel approach to derive functionally mature human liver organoids from pluripotent stem cells (PSCs), including human embryonic stem cells and iPSCS [106]. Transcriptome analysis and functional assays were used to verify organoid maturity. The generated organoids can be applied in drug toxicity prediction and screening.

dECM can not only be used as a scaffold for organoids; the various cytokines within dECM can also promote organoid formation [107]. Saheli et al. demonstrated that the organoids cultured in the presence of 3D liver dECM gel showed the epithelial phenotype of hepatocytes with a higher cell viability and significant upregulation of hepatocyte-specific transcripts and functions compared to hydrogel-free organoids or organoids in solubilized dECM, as well as 3D collagen gel organoid and traditional 2D culture [108]. Besides, the ECM can not only carry some biological information but also provide mechanical properties necessary for organoid construction. The mechanical properties of dECM depend on factors such as the concentration, composition, and source of dECM solution. Therefore, when developing dECM for organoids, appropriate decellularization methods should be selected to retain specific ECM components for special organoids and reduce the variation between batches. In addition, the mechanical properties of dECM gels, such as storage modulus, loss modulus, elastic modulus, and hardness, should be examined [109].

Combining organoids with 3D bioprinting can help to develop spatial structures that are more suitable for organoid growth [110]. Moreover, 3D bioprinting can produce vessels of various sizes, allowing the construction of larger organoids [111]. However, 3D bioprinted organoids have a good potential for application in precision medicine and regenerative medicine. However, it is important to address safety, ethical, and legal issues before clinical application [112,113].

### 6.4. Personalized Medicine

Treatment options for diseases tend to target a certain patient group, and each patient’s disease state is caused by complex factors. For some patients with rare diseases or tumor diseases, they need more personalized treatment. Precision medicine develops a personalized treatment plan based on a patient’s physiological genetic characteristics and drug response [114]. Therefore, patients can receive optimal treatment with fewer adverse effects and improved prognosis.

3D bioprinting can create complex and reproducible 3D structures, which has important implications for personalized medicine. For example, if tumor cells, stromal cells, extracellular matrix proteins, and other components from a patient are used for 3D bioprinting, a 3D tumor model for that patient can be created and used as a screening tool for various therapies before clinical treatment to find the right treatment for this patient for personalized treatment. The use of patient-derived tumor cells to construct a tumor microenvironment is, therefore, more personalized than the use of 3D tumor models derived from cell lines.

Xie et al. used HepaRG cells and 3D bioprinting to construct liver organoids that preserved liver function and prolonged survival in mice with liver failure after abdominal transplantation [101]. They also developed an individualized HCC model by extending the modeling system. The bioink is composed of gelatin, sodium alginate, and primary HCC cells isolated from surgical specimens. They successfully created a 3D bioprinted model constructed from the patient’s own cells, which grew well and could be used for long-term culture. Moreover, the constructed model retains the HCC characteristics of the original patient and can intuitively and quantitatively display the results of drug screening for personalized treatment. Table 2 summarizes the liver 3D bioprinting in different fields of the application.

## 7. Current Challenges and Future Perspectives

In the past few decades, decellularization technology has developed rapidly, from the initial decellularized simple tissue scaffolds to the whole organ decellularized scaffolds. Physical, chemical, and enzymatic treatments can remove the immunogenic cellular components of dECM scaffolds, retain the effective components and structure, and provide a suitable microenvironment for tissue repair and regeneration.

3D bioprinting is a fast-growing area of biomaterials engineering that can push biomedical engineering forward [115]. Three-dimensional bioprinted scaffolds can provide spatial depth and better intercellular communication for cells, which can better simulate the in vivo environment and help to reduce the dependence on animal experiments. Batch-to-batch consistency also facilitates drug screening. Three dimensional bioprinting has promoted the application of tissue and organ constructs, drug screening, and organoid model systems, making it possible to produce functional human organs, such as the liver, heart, and skin, on a large scale in the future.

The liver model constructed by the combination of 3D bioprinting and dECM has many important implications: (1) It can simulate various human liver diseases in vitro to explore the underlying molecular mechanisms of diseases; (2) to reveal the effects of cell-cell and cell-ECM interactions on hepatocyte function; (3) accurately predict clinical trial results to avoid drug development failure due to hepatotoxicity; (4) It is helpful for the construction of artificial liver for clinical transplantation.

However, there are several challenges in translating 3D bioprinted dECM structures loaded with cells from laboratory studies to clinical applications:

Despite the rapid development of dECM bioink in the field of in vitro printing, there are still some problems that need to be optimized before its clinical application. For example, dECM does not dissolve in vivo culture, or the degradation rate is consistent with tissue regeneration; It needs to be nontoxic, bind to cells, and allow angiogenesis. If we want to ensure that the cells in the bioink are not damaged in 3D bioprinting, then the appropriate proportion of bioink and cell source, as well as standardized printing technology, are needed.

An important problem facing clinical translation is vascularized tissues and organs [116]. Angiogenesis is essential for cell proliferation. However, the hierarchically complex vascular network from arteries and veins to capillaries is difficult to replicate. There are two approaches to overcoming this problem. One approach is to embed microchannels to increase the diffusion of oxygen and nutrients [117]. Another approach is the patterning of angiogenic growth factors or cells in printed constructs to promote vascular development after transplantation [118]. Moreover, the long-term immunogenicity and compatibility of the implanted 3D-printed tissues with the recipient tissues have not been determined [119].

The current research has proved that the dECM mixed with other materials, such as gelatin, can increase the printability and mechanical properties [120]. Therefore, by mixing dECM with other materials, bioinks with more advantages and better performance can be created for the 3D bioprinting of liver tissue in the future. Researchers should aim for more precise cell deposition, increased vascularization, and innervation. Many challenges remain in the field before large-scale 3D bioprinting can be implemented. If these challenges can be overcome, clinical application of 3D printed structures with dECM as bioink will be realized in the future.

## 8. Conclusions

In this review, we introduce the structure, origin, properties, and functions of liver dECM, as well as the specific ways of combining liver 3D bioprinting and dECM and their applications in multiple fields. Liver 3D bioprinting and dECM are widely used in the field of tissue engineering, but there are few reports on the combination of the two. Researchers still have some problems to solve in combining the two and playing their respective advantages better.

## Figures and Tables

**Table 1:** The mechanisms, advantages, and disadvantages of various decellularization methods.

Method	Mechanisms	Advantages	Disadvantages	Reference
Physical treatments				
Freeze-thaw cycles	Intracellular ice crystals are formed, which decompose the cell membrane and promote cell lysis.	It improves the efficiency of decellularization and reduces the residue of chemicals.	The cellular contents need to be removed later.	[7,22,23,38,41]
Perfusion	Whole organs/tissues were cannulated and decellularized by rapid perfusion of the entire vasculature.	It is suitable for decellularization of whole organs with vascular systems.	Perfusion requires additional hardware and sophisticated flow control devices.	[7,22,23,38,41]
Immersion and agitation	It refers to the method of immersing tissue in decellularized solution with continuous mechanical agitation.	It is suitable for small, fragile organ sections and tissues without innate vascular structures. It is easy to obtain and perform.	Efficiency is affected by agitation intensity, decellularization agent, and tissue size.	[7,22,23,38,41]
Chemical treatments				
Ionic detergents	Lyse the nuclear and cytoplasmic membranes by disrupting lipid-lipid, lipid-protein, DNA-protein, and protein-protein interactions. For example: sodium dodecyl sulfate (SDS).	They can completely remove natural cells and genetic materials.	They have harmful effects on ECM structures and bioactive components and is difficult to remove.	[7,22,23,38,41]
Non-ionic detergents	Lyse cell membranes and dissociate DNA from proteins without disrupting protein interactions. For example: Triton-100.	They are gentler than ionic detergents and do not destroy the structure and orientation of collagen.	Remove cells incompletely.	[7,22,23,38,41]
Acids	Disrupt cell membranes, solubilize cytoplasmic organelles, induce cell lysis. For example: peracetic acid.	They have bactericidal activity against a variety of bacteria, viruses and fungi.	Disrupt ECM microstructure, reduce collagen content and weaken tissue strength.	[7,22,23,37,38,41]
Bases	Decellularized tissues by denaturing chromosomal DNA and inducing cell lysis. For example: sodium hydroxide.	They can efficiently remove cellular remnants.	They affect ECM structure and reduce GAG and growth factor content.	[7,22,23,37,38,41]
Hypertonic solutions	Cell lysis and cell death are induced by osmotic effects.	Efficient protein removal.	They are unable to completely eliminate cell remnants.	[7,22,23,37,38,41]
Hypotonic solutions	Cell lysis and cell death are induced by osmotic effects.	Efficient removal of nuclei and DNA.	They are unable to completely eliminate cell remnants.	[7,22,23,37,38,41]
Enzymatic treatments				
Nuclease	Catalyze the hydrolysis of deoxyribonucleotides and ribonucleotide chains.	Efficient removal of nucleic acids.	Results in the loss of ECM components such as GAG, laminin. The residual may elicit an immune response.	[7,22,23,41]
Trypsin	Dependent on the breakage of carbon side of carboxyl-side of arginine and lysine, resulting in the separation of cellular components from the ECM.	Without cytotoxic effect.	It may result in ECM damage and altered mechanical stability.	[7,22,23,41]

**Table 2:** Summary of the application of liver 3D bioprinting in different fields.

Cell Sources	Biomaterials	Shape/Structure	Manufacturing Strategy	Research Applications	Reference
HepaRG cells	Alginate, gelatin and human extracellular matrix (hECM)	Grid	Extrusion-based bioprinting	Study virus biology and develop new antiviral compounds	[90]
Human-induced hepatocytes (hiHep cells)	GelMA, liver dECM	Gear-like	DLP-based bioprinting	Liver microtissue fabrication	[93]
HepG2 cells	GelMA, liver dECM	Hexagonal constructs	Rapid light-based 3D bioprinting	Tunable mechanical properties for in vitro disease modeling	[94]
HLCs, HUVECs, HHSCs	Liver dECM	Sinusoidal lumen-like network	Extrusion-based bioprinting	Drug development and screening	[98]
Primary HCC cells	Gelatin, sodium alginate	Grid	Uncertain	Predict patient-specific drugs for personalized treatment	[101]
HepaRG cells	Sodium alginate, dipeptide-based bioink	Biomimetic lobule structure	Droplet-based bioprinting	In vitro construction of liver organoids with biomimetic lobule structure	[104]
